# Electron Transfer Strategies to Regulate Carriers’ Separation for Intensive Pyroelectric Dynamic Therapy With Simultaneous Photothermal Therapy

**DOI:** 10.3389/fchem.2022.874641

**Published:** 2022-04-12

**Authors:** Bingxia Sun, Yun Meng, Tianlin Song, Jieyun Shi, Xinhong He, Peiran Zhao

**Affiliations:** ^1^ Shanghai Key Laboratory of Green Chemistry and Chemical Processes, School of Chemistry and Molecular Engineering, East China Normal University, Shanghai, China; ^2^ Tongji University Cancer Center, Shanghai Tenth People’s Hospital, Tongji University School of Medicine, Shanghai, China; ^3^ Department of Interventional Radiology, Fudan University Shanghai Cancer Center, Shanghai, China; ^4^ Department of Materials Science and State Key Laboratory of Molecular Engineering of Polymers, Fudan University, Shanghai, China

**Keywords:** Au–SnSe–PVP nanomaterials, photothermal therapy, pyroelectric dynamic therapy, separation of electrons and holes, hydroxyl radical

## Abstract

Endogenic heat shock proteins and uneven local heat distribution are two main problems in traditional tumor hyperthermia therapy strategies. Aiming at solving these problems, we designed Au–SnSe–PVP nanomaterials (ASNPs) by modifying Au nanoparticles (Au-NPs) and biocompatible PVP on SnSe nanorods *via* a new reactive oxygen species production strategy. The ASNPs with excellent photothermal conversion performance can produce thermoelectric effects in response to temperature differences during photothermal conversion. The modification of Au-NPs can attract free electron (e^–^) to accumulate and promote the separation of e^–^ and holes (h^+^) in the thermoelectric process, thereby further promoting e^–^-rich Au-NPs-induced H_2_O_2_ homolysis and h^+^–H_2_O half-reaction to generate hydroxyl radicals, realizing the synergistic application of photothermal therapy and pyroelectric dynamic therapy in tumor treatment.

## Introduction

The application of heat as energy in tumor treatment, for example, hyperthermia, which is a treatment method that kills tumor cells *via* heat application locally to the tumor areas, results in dehydration, protein denaturation, and even coagulative necrosis. Hyperthermia has been widely used in tumor treatment in recent years due to its advantages of easy access to energy sources, noninvasiveness or minimally invasiveness, and high curative effects. For example, photothermal therapy (PTT) (Dong et al., 2018; Li et al., 2019; Sun et al., 2020; Wang et al., 2020b; Simon et al., 2021) and magnetocaloric therapy (MTT) (Alphandery et al., 2017; Albarqi et al., 2019; Das et al., 2019; Qian et al., 2020) of tumors have attracted considerable attention in fundamental research, and high-intensity focused ultrasound (HIFU) (Izadifar et al., 2020), microwave ablation (MWA), and radiofrequency ablation (RFA) have entered clinical application (Meloni et al., 2017; Palussiere et al., 2017; Sidoff and Dupuy, 2017; Cho et al., 2020; Hasegawa et al., 2020; Maeda et al., 2020; Sparchez et al., 2020; Teng et al., 2020; Yang et al., 2020). However, there are still two main obstacles that stand in the way of the development of traditional hyperthermia methods. On the one hand, the therapeutic effect is easily disturbed by the high expression of heat shock proteins (HSPs) (Chen et al., 2017; Gao et al., 2020). On the other hand, during hyperthermia, the local temperature at the tumor region is not always high enough to destroy tumor cells without causing damage to surrounding normal tissues, resulting in potential possibilities of tumor recurrence and metastasis. Therefore, developing new strategies to solve these problems is highly desired but is challenging.

However, as one of the basic energy forms, heat is not only a type of energy to attack cells but also a type of field to excite physical effects or chemical reactions. Recently, as a type of excitation field, heat has been widely used in tumor treatment, including thermosensitive polymers as carriers for drug delivery (Li et al., 2015; Wang et al., 2017) and thermochromic materials as indicators for tumor hyperthermia monitoring (Shen et al., 2020). Notably, based on the thermoelectric semiconductor effects, we had proposed the pyroelectric dynamic therapy (PEDT), which generated hydroxyl radicals (∙OH) for HSPs attack, tumor cells killing, and enhanced tumor hyperthermia treatment (Tang et al., 2018). Thermoelectric semiconductor nanomaterials can be excited by temperature difference to generate free e^–^ and h^+^, which further initiates h^+^–H_2_O and e^–^–O_2_ reactions to generate reactive oxygen species (ROS) such as ∙OH and ∙O_2_^−^. Generally, to adapt to *in vivo* application and better respond to the temperature changes in the tumor area, thermoelectric semiconductor nanomaterials with good biosafety, excellent thermoelectric performance, and appropriate valence/conduction band potentials (valence band potential greater than ∙OH/H_2_O or ∙OH/OH^−^redox potential or conduction band potential lower than ∙O_2_^−^/O_2_ redox potential) can be promisingly used in PEDT. Furthermore, PEDT is favorable in combination with some hyperthermia methods, such as PTT, MTT, MWA, and RFA, to generate ROS and leads to direct tumor cells apoptosis or heat resistance inhibition, further enhancing the therapeutic effect of hyperthermia. Unfortunately, owing to the low carrier mobility, low e^–^–h^+^ separation efficiency in nanosemiconductors and low h^+^–H_2_O half-reaction efficiency, the effect of PEDT is extremely limited. Solving the aforementioned scientific problems with the material design is crucial for tumor hyperthermia treatment.

In this study, we designed Au–SnSe–PVP nanomaterials for PEDT–PTT synergy tumor therapy. Considering the biosafety, temperature difference response ability, and ROS generation requirements, SnSe was selected as PEDT nanomaterials for the advantages of good biosafety, good photothermal conversion performance, superior thermoelectric efficiency, and appropriate valence/conduction band potentials. After combining Au nanoparticles onto the surface of SnSe, the photothermal conversion property was improved, and the migration and separation properties of e^–^ and h^+^, which was activated by thermoelectric effects, could be significantly improved due to the potential difference between electron acceptor Au-NPs and SnSe nanosemiconductors, resulting in promoted h^+^–H_2_O half-reaction efficiency (Wang et al., 2020a; Zhang et al., 2020; Wang et al., 2021). Moreover, e^–^-rich Au-NPs could catalyze tumor endogenous H_2_O_2_ into ∙OH with high reactivity, together with h^+^–H_2_O-induced ·OH generation. The technique can not only attack HSPs for more efficient PTT but also destroy tumor cells under temperatures that would not have been fatal with PEDT, providing a paradigm of the material design strategy for efficient tumor hyperthermia therapy.

## Materials and Methods

### Reagent

The selenium powder (99.5%, 325 mesh, Alfa Aesar), sodium ascorbate (99%, Adamas-Beta), PVP (MW = 40,000, K30, Sigma), SnCl_2_·2H_2_O (99.99%), NaBH_4_ (98%), and HAuCl_4_·3H_2_O (99.9%) were purchased from Aladdin Biochemical Technology Co., Ltd. (Shanghai, China). Methanol (GR) and K_2_CO_3_ (AR) were purchased from Sinopharm Chemical Reagent Co., Ltd. Luciferase isothiocyanate (FITC, ≥90%), 3, 3′, 5, 5′-tetramethylbenzidine (TMB), and methylene blue (MB, 95%) were purchased from Sigma-Aldrich Co., Ltd. DCFH-DA and Hoechst 33342 staining solution were purchased from Beyotime Co., Ltd. All chemical agents in this work were utilized without further purification.

### Synthesis of SnSe Nanorods

First, an A stock Se precursor solution was prepared. In brief, 0.104 g Se powder was added into a 100-mL beaker filled with 40 mL deionized (DI) water, heated in advance at 60°C, and then stirred vigorously for 15 min. When the Se powder floated on the upper surface of the water, 0.20 g NaBH_4_ was added and stirred for another 15 min until the solution became transparent. Then, the B stock Sn precursor solution was prepared by adding 0.20 g SnCl_2_·2H_2_O into 24 mL of 24 mg/mL sodium ascorbate solution with stirring until the solution turned milky white. Finally, A and B were mixed and transferred to a 100-mL autoclave vessel and then reacted at 200°C for 20 h. The products were washed with DI water three times and then dried at 60°C to obtain the SnSe nanorod powder.

### Synthesis of Au–SnSe

For the synthesis of Au–SnSe, 10 mL of 20 mM HAuCl_4_ solution and 10 mL of 20% methanol were added to the 10 mL of 1 mg/mL SnSe aqueous dispersion system. The pH of the solution was adjusted to 9.7 with K_2_CO_3_ and then sonicated (40 kHz, 80 W) for 1 h. Finally, the products were washed with DI water three times to obtain Au–SnSe.

### PVP Modification

For PVP modification, 100 mg Au–SnSe and 0.5 g PVP were dispersed in 100 mL DI water and sonicated at 60°C for 3 h. After being centrifugated with DI water three times, ASNPs were obtained.

### Characterization

Transmission electron microscopy (TEM) images were collected using JEOL 200CX at 300 kV. X-ray diffractometry (XRD) measurement was performed on a Rigaku D/MAX-2250 V X-ray diffractometer at Cu Kα (*λ* = 0.154056 nm) within the 2θ range of 20–90° at a scanning rate of 5°/min. Fourier transform infrared (FT-IR) spectra were obtained using a Bruker Tensor II FTIR spectrometer. X-ray photoelectron spectroscopy (XPS) plots were detected by Shimadzu, AXIS SUPRA. Zeta-potential data were collected with Nicomp Z3000 SOP. Ultraviolet-Visible (UV-Vis) absorption spectra were recorded on Shimadzu UV-3600 Plus. An infrared thermal imager (FLIR A325SC camera) was used to record temperature changes and thermal image information. MTT assays were conducted with a Spark™ multimode microplate reader. Confocal laser scanning microscopy (CLSM) images were obtained using a Nikon A1+R-980 confocal microscope.

### Measurement of ASNPs Photothermal Performance

The photothermal performances of SnSe–PVP and ASNPs were measured using the infrared thermal imager under 808-nm laser irradiation. The solution temperature was recorded under SnSe–PVP and ASNPs aqueous (1 mL, 50 μg/mL) with different 808-nm laser power densities (0.5, 1, and 1.5 W/cm^2^) and 808-nm laser (1 W/cm^2^) with different SnSe–PVP and ASNPs concentrations (100, 50, 25, and 12.5 μg/mL). Then, the ASNPs aqueous dispersion was subjected to multiple laser irradiation heating and natural cooling cycles, and the temperature curve was recorded to measure the photothermal stability and calculate the photothermal conversion efficiency.

### Determination of ∙OH

A total of two typical ∙OH indicators, MB, and TMB were used to detect ∙OH. First, SnSe–PVP and ASNPs were dispersed in MB solution at a concentration of 50 μg/mL. The as-achieved solution was then irradiated with an 808-nm laser (1 W/cm^2^) for 5 min, then cooled naturally, and cycled different times (1, 2, and 4 times). Subsequently, SnSe–PVP and ASNPs were dispersed in H_2_O and H_2_O_2_ at a concentration of 50 μg/mL, then irradiated with an 808-nm laser (1 W/cm^2^) for 5 min, and cooled naturally. The UV-Vis spectra of MB were measured using a UV-Vis spectrophotometer. Similarly, TMB was also used to further detect the ∙OH productivity of ASNPs with different laser power densities and material concentrations.

### Cell Culture

The 4T1 murine breast adenocarcinoma cell line (obtained from the Shanghai Institute of Cells, Chinese Academy of Sciences) was cultured in RPMI 1640 (Gibco, United States) supplemented with 10% heat-inactivated fetal bovine serum, streptomycin (100 mg/mL), and penicillin (100 units/mL) and cultured at 37°C in a humidified atmosphere along with 5% CO_2_.

### Cytotoxicity of ASNPs and Phototoxicity

Cytotoxicity and phototoxicity were tested on 4T1 cells. The 4T1 cells were seeded into 96-well culturing plates in a density of 5×10^3^ cells/well and incubated overnight. Next, ASNPs dissolved in the fresh culture medium with the concentrations of 0, 12.5, 25, 50, and 100 ppm were added to exchange the culture medium in the 96-well plates. After a 6 h co-incubation, the culture medium was refreshed with the fresh culture medium, then irradiated with an 808-nm laser (1 W/cm^2^) for 5 min, and incubated for another 24 h. Another 96-well plate inoculated with 4T1 cells was irradiated with an 808-nm laser at different power densities (0.5, 1, 1.25, and 1.5 W/cm^2^) for 5 min without ASNPs and then cultured for another 24 h. The cell viability was examined *via* a standard MTT assay on a microplate reader (Bio-TekELx800, United States) by testing the absorbance at 490 nm.

### Confocal Fluorescence Imaging

The 4T1 cells were seeded with 1×10^5^/mL cells in RPMI 1640 in confocal laser scanning microscopy (CLSM) culture vessels and permitted to adhere overnight.

The 4T1 cells were cocultured with FITC-labeled ASNPs for 4 or 6 h and then washed with PBS. The endocytosis was observed under confocal mode after Hoechst 33342 staining.

The 4T1 cells were first cocultured with ASNPs (50 μg/mL) for 6 h. After refreshing the culture medium, the cells were irradiated with an 808-nm laser (1 W/cm^2^) for 5 min and then incubated for another 12 h. The cells were stained with Hoechst 33342 as nuclear staining solution and DCFH-DA as the ROS fluorescent probe before CLSM observation.

### Biosafety Evaluation of ASNPs *In Vivo*

All animal experiments were conducted according to the guidelines of the Institutional Animal Care and Use Committee and the Experimental Animal Ethics Committee of East China Normal University, and the accreditation number was m^+^R20190701.

ICR mice (female, 7 weeks old) and BALB/c mice (female, 6 weeks old) were obtained from the Laboratory Animal Center of East China Normal University.

A total of fifteen ICR mice were randomly divided into three groups. The control group was intravenously (*i.v.*) injected with 100-μL saline solution, 3 and 30-days groups were *i.v.* injected with 100-μL ASNPs solution (30 mg/kg). The mice in the control and 30-days groups were weighed every 3 days. Three and 30 days after injection, one mouse from each group was randomly selected and killed to take major organs (heart, liver, spleen, lung, and kidney) for further histopathology analysis using a typical hematoxylin and eosin (H&E) staining assay. Meanwhile, the blood samples of the other mice in each group were collected for routine blood and biochemical analysis.

### Antitumor Performance of PTT–PEDT *In Vivo*

The BALB/c mice were subcutaneously inoculated with 4T1 cells (1×10^6^, suspended in 100 μL PBS) on the right posterior axilla. When the tumor size reached 200 mm^3^, the mice were randomly divided into four groups: control group, ASNPs group (*i.v.*, 15 mg/kg), near-infrared (NIR) group (808 nm laser, 1 W/cm^2^, 5 min), and NIR + ASNPs group (*i.v.*, 15 mg/kg; 808 nm laser, 1 W/cm^2^, 5 min), with six mice in each group. One day after treatment, one mouse in each group was randomly selected and killed, and the tumors were taken for H&E and TUNEL immunofluorescence staining. The body weight and tumor volume [V= (length × width × width)/2] of the remaining mice were recorded every other day. The mice were euthanized when the tumor volume reached the ethical value.

## Results and Discussion

### Characterization of ASNPs

**FIGURE 1:**
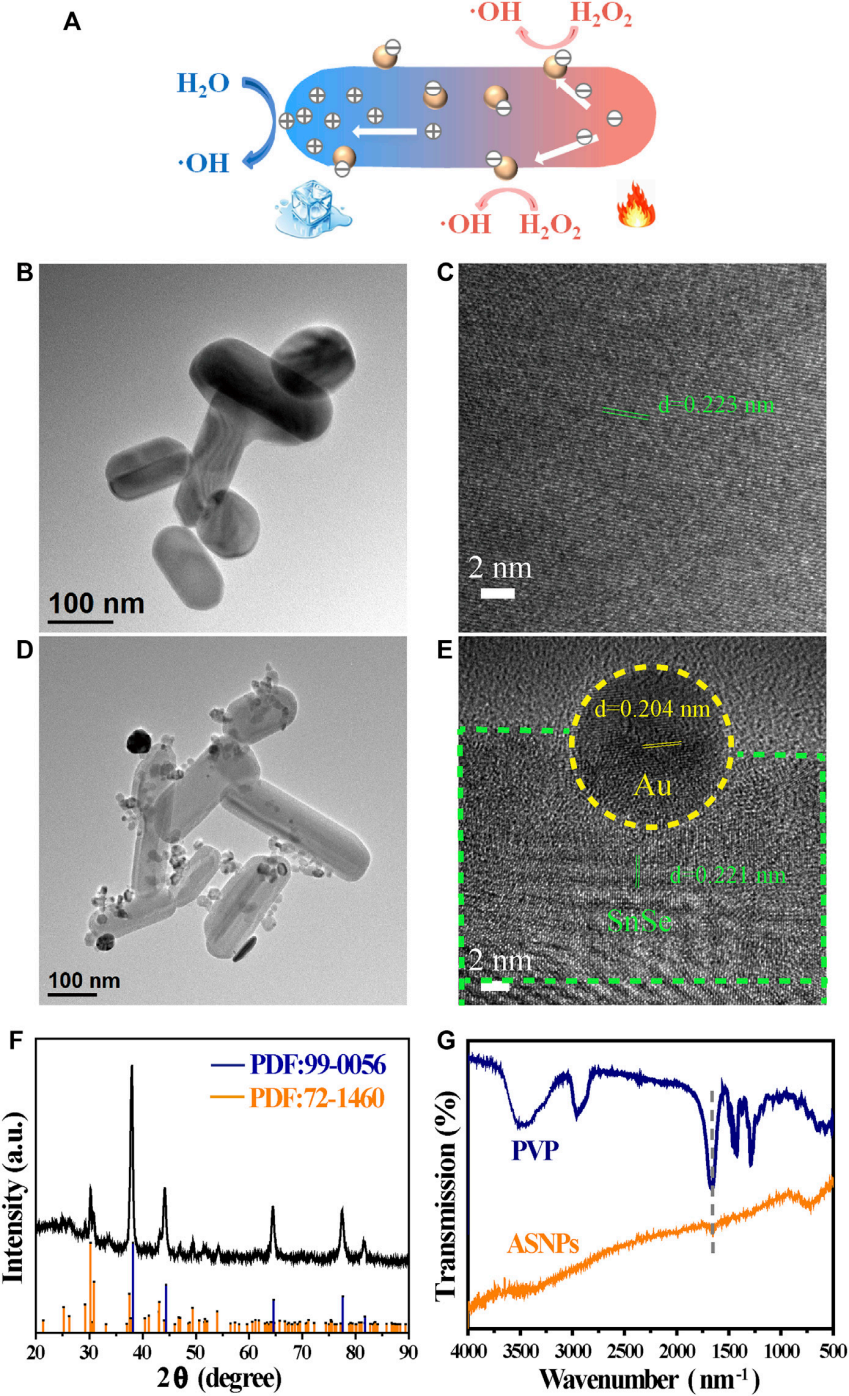
Description and characterization of SnSe and ASNPs. **(A)** Schematic illustration of ASNPs generating ∙OH in the PEDT process. **(B)** TEM and **(C)** HRTEM images of SnSe. **(D)** TEM and **(E)** HRTEM images of ASNPs. **(F)** XRD patterns and **(G)** FT-IR spectra of ASNPs.

**FIGURE 2:**
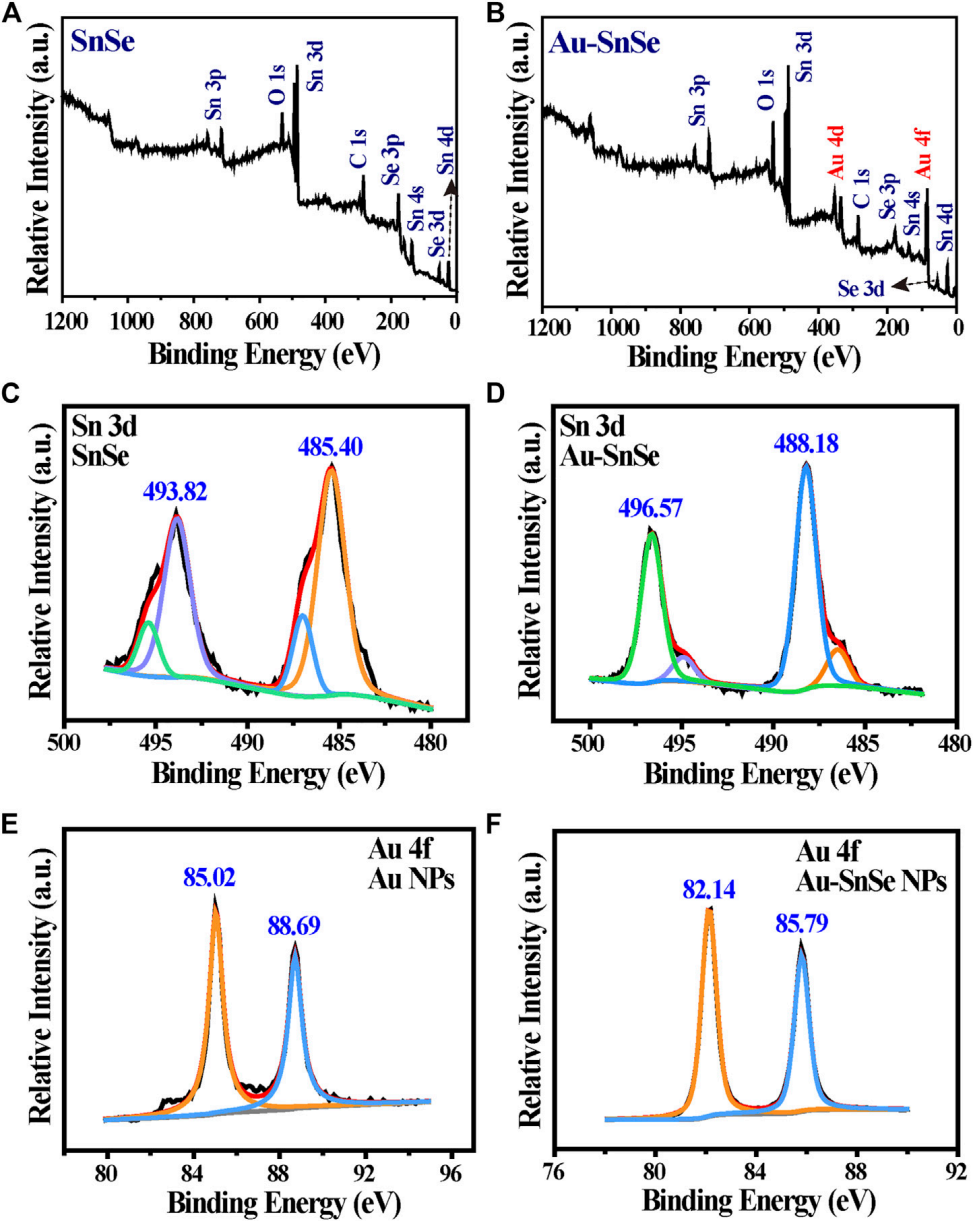
Representative XPS spectra of **(A)** SnSe, **(B)** Au–SnSe, **(C)** Sn 3d of SnSe, **(D)** Sn 3d of Au–SnSe, **(E)** Au 4f of SnSe, and **(F)** Au 4f of Au–SnSe.

Figure 1A is a schematic of ASNPs generating ∙OH in the PEDT process. Upon the irradiation of the 808-nm laser, heat was generated along the nanorod, and the temperature difference was formed in the heat conduction process. Under a temperature difference, the carriers (e^–^ and h^+^) in SnSe could be activated and driven to the hotter and colder sides, respectively. Owing to the differences of potential between electron acceptor Au-NPs and SnSe, the e^–^ accumulated on Au-NPs, hence promoting the carriers’ mobility and e^–^ and h^+^ separation, facilitating the gathering of h^+^ at the low-temperature end of ASNPs. This resulted in a significant ∙OH productivity increase due to e^–^-rich Au causing H_2_O_2_ splitting (Lv et al., 2020) and h^+^–H_2_O reactions. The TEM images (Figures 1B,D) show that the average particle size of SnSe and ASNPs is approximately 200 and 250 nm, respectively. The high-resolution TEM (HRTEM) images (Figures 1C,E) indicate considerable changes in interplanar spacing of SnSe and ASNPs, suggesting a distinct lattice distortion in ASNPs induced by the modification of Au-NPs. XRD patterns show that Au–SnSe composite structures were successfully constructed (Figure 1F). The FT-IR spectra (Figure 1G) and zeta-potential (Supplementary Figure S1) results show that PVP was successfully modified onto the surface of Au–SnSe. In Figure 2, XPS was used to characterize the electron transfer of Au–SnSe compared with SnSe (Figures 2A–F). The binding energies of the Sn 3d and Au 4f peaks in Au–SnSe shifted to the high and low fields, respectively, after the construction of Au-NPs, indicating that electrons transferred from SnSe to Au, thereby proving the enhanced separation of e^–^ and h^+^ property of Au–SnSe nanocomposites.

### ASNPs Photothermal Conversion Performance

**FIGURE 3:**
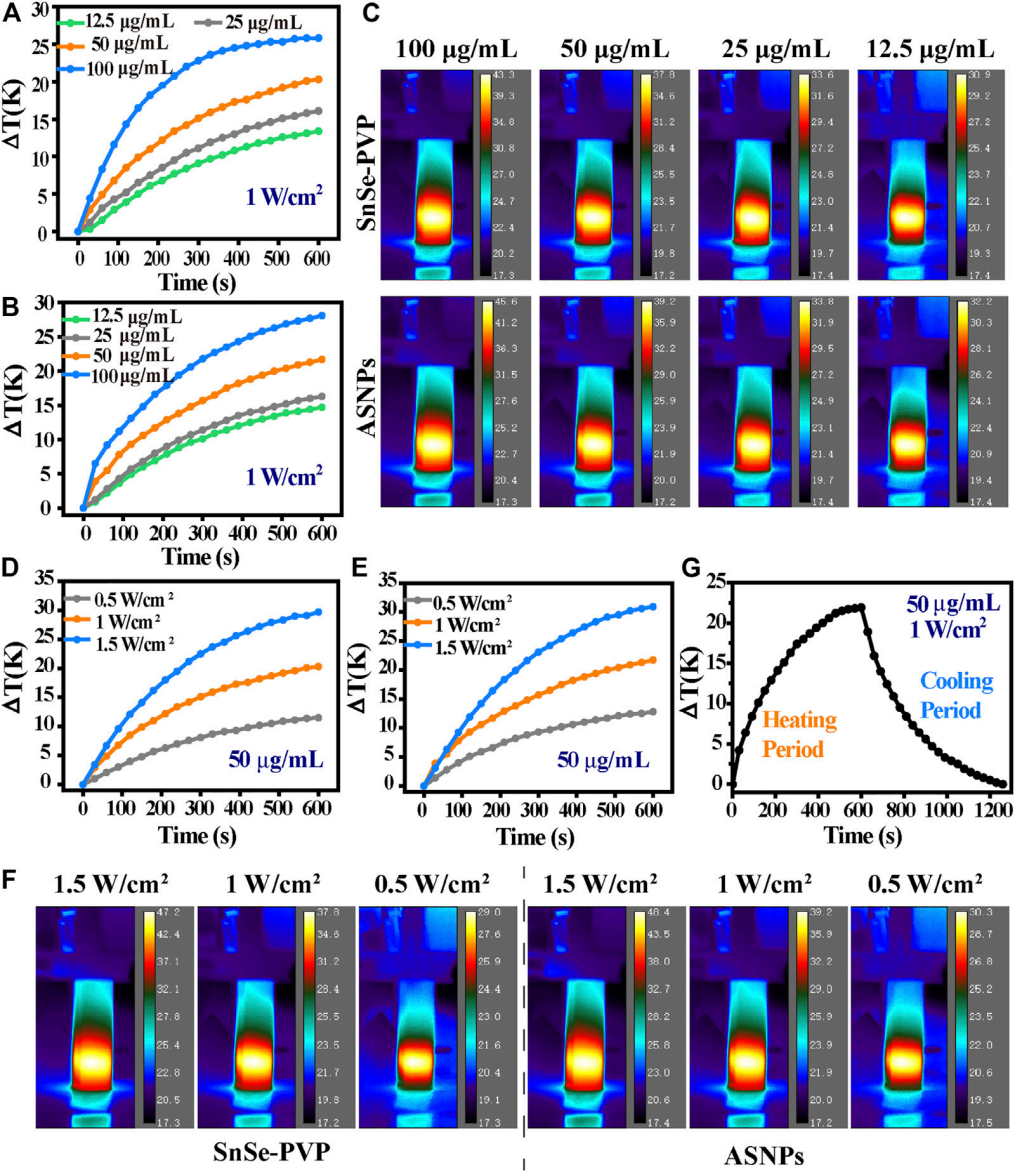
Characterization of photothermal conversion properties. Temperature curve as a function of time of **(A)** SnSe–PVP, **(B)** ASNPs solutions (12.5, 25, 50, and 100 μg/mL) with 808-nm laser irradiation (1 W/cm^2^), and **(C)** the corresponding thermal imaging figure at the maximum stable temperature. Temperature curve as a function of time of **(D)** SnSe–PVP, **(E)** ASNPs solutions (50 μg/mL) with 808-nm laser irradiation at various laser intensities (0.5, 1, and 1.5 W/cm^2^), and **(F)** corresponding thermal imaging figure at the maximum stable temperature. **(G)** Temperature curve as a function of time of ASNPs solution (50 μg/mL): under 808-nm laser irradiation (1 W/cm^2^) to a maximum stable temperature (heating period); naturally cooling to a stable temperature without laser irradiation (cooling period).

Figures 3A–F show the temperature change of SnSe–PVP and ASNPs under different material concentrations and 808-nm laser intensities. The temperature change is proportional to the material concentration and laser intensity, and the photothermal conversion ability of ASNPs is slightly better than that of SnSe–PVP under the same conditions. The temperature variation curves of ASNPs were recorded under irradiation of an 808-nm laser at 1 W/cm^2^ and in the process of natural cooling (Figures 3G, Supplementary Figure S2). These results indicated that ASNPs had stable photothermal conversion ability. By measuring the UV-Vis-NIR absorption spectra of ASNPs aqueous dispersion, the extinction coefficient of ASNPs at 808 nm was calculated to be 12.41 L∙g^−1^∙cm^−1^ (Supplementary Figures S3, S4). According to the aforementioned experimental results and calculation methods in previous literature reports (Lyu et al., 2016), the photothermal conversion efficiency of ASNPs was estimated to be 32.62%.

### Determination of ∙OH Production in PEDT Processes

**FIGURE 4:**
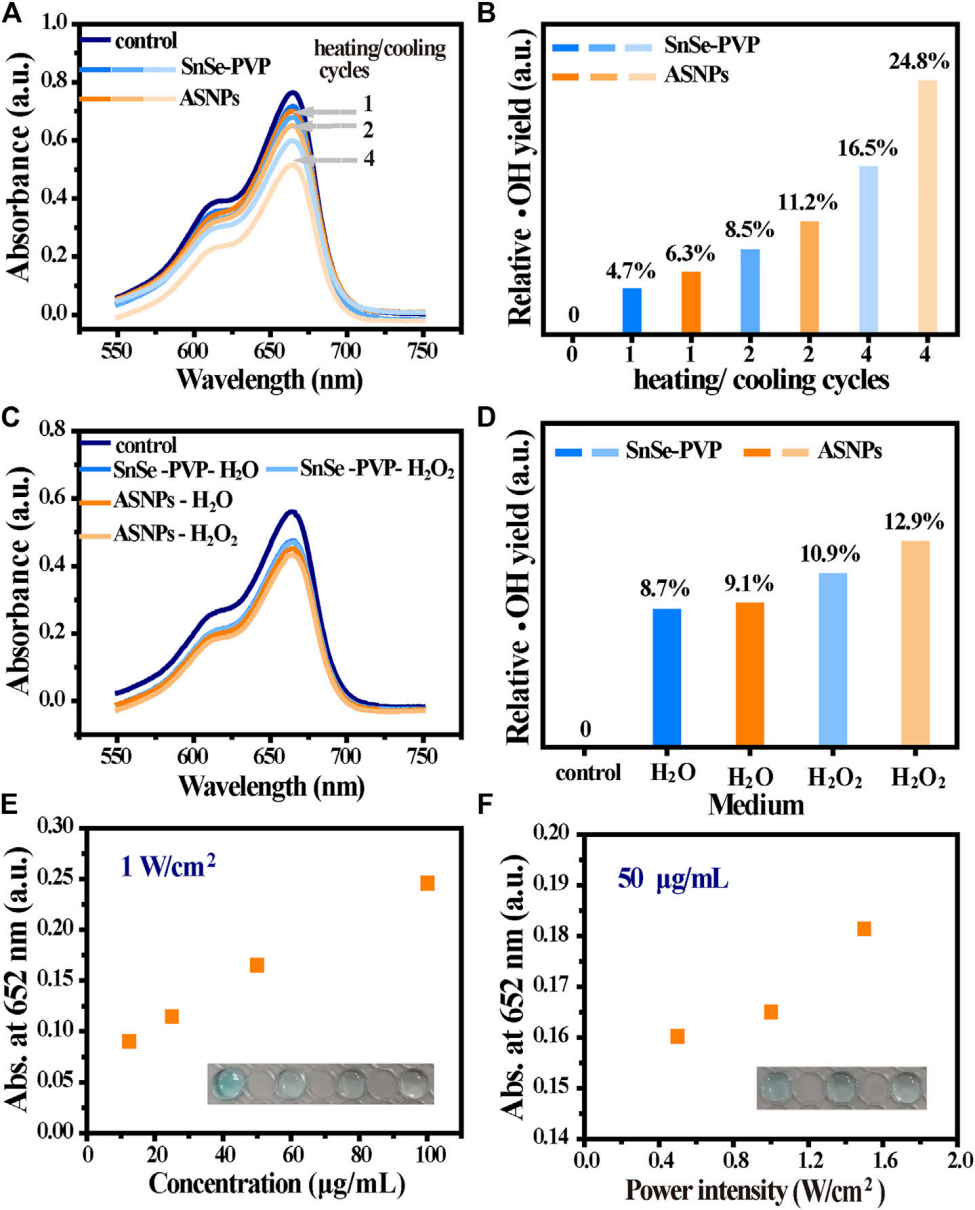
∙OH generation performance in the PEDT process. **(A)** MB degradation under different heating/cooling cycles (1, 2, and 4 times) for SnSe–PVP and ASNPs solutions (50 μg/mL) at a power intensity of 1 W/cm^2^ and **(B)** the corresponding relative ∙OH yield. **(C)** Effect on MB degradation for dispersed SnSe–PVP and ASNPs in H_2_O and H_2_O_2_ (50 μg/mL) with a power intensity of 1 W/cm^2^ for one heating/cooling cycle and **(D)** the corresponding relative ∙OH yield. The absorbance change at 652 nm after treating TMB with **(E)** different concentrations of ASNPs (12.5, 25, 50, and 100 μg/mL) and **(F)** laser intensities (0.5, 1, and 1.5 W/cm^2^).

Compared with SnSe–PVP, ASNPs exhibited superior MB degradation ability, indicating higher ∙OH productivity of ASNPs upon 808-nm laser irradiation (Figures 4A,B). Furthermore, no obvious ∙OH production differences were observed in the H_2_O or H_2_O_2_-dispersed SnSe–PVP group, whereas the ASNPs group presented a higher ∙OH productivity in H_2_O_2_ media (Figures 4C,D). The aforementioned results further confirmed that Au-NPs could conduct e^–^ out of ASNPs and promoted the separation of e^–^ and h^+^ in the thermoelectric process, and the e^–^-rich Au-NPs could catalyze H_2_O_2_ into ∙OH. Thus, the reaction efficiency of h^+^–H_2_O half-reaction was improved, and the e^–^-rich Au-NPs-induced H_2_O_2_ homolysis process was constructed, thereby significantly improving PEDT performance. In addition, TMB was used to test the influence of ASNPs concentrations and laser power densities (resulting in different temperature differences) on the ∙OH yield, and the results showed that both of them are positively correlated with ∙OH yield (Figures 4E,F).

### Therapeutic Efficacy of ASNPs *In Vitro*

**FIGURE 5:**
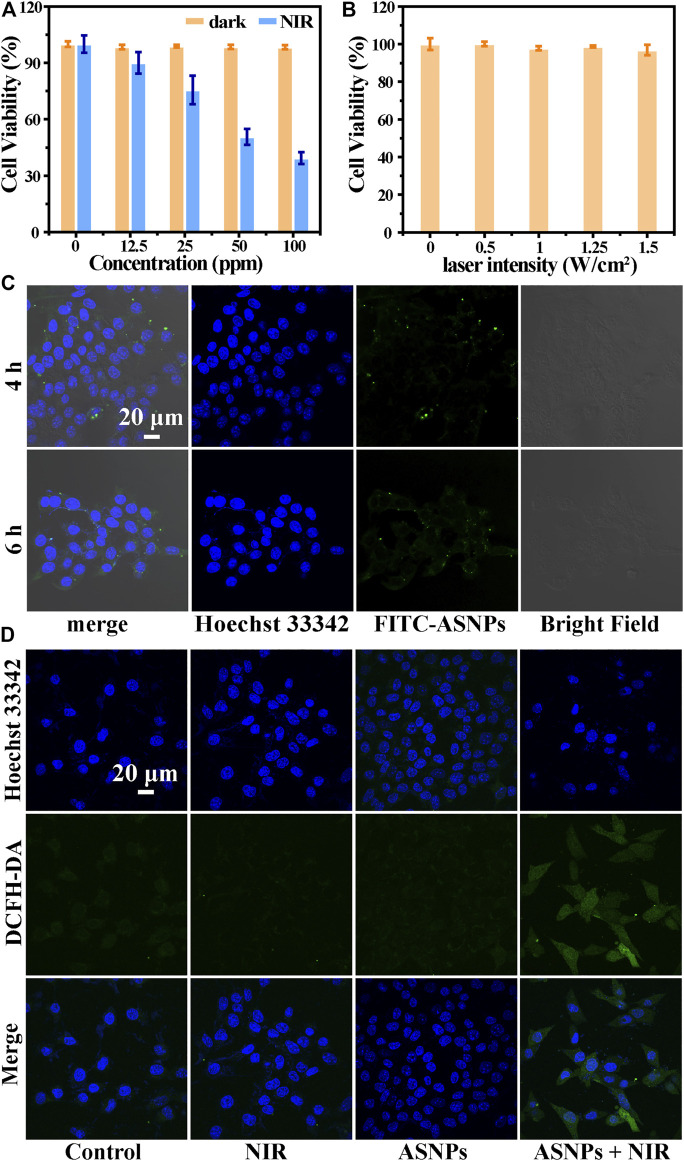
**(A)** Cell viability of 4T1 cells coincubated with different concentrations of ASNPs with or without NIR irradiation (*n* = 5, mean ± SD). **(B)** Cell viability of 4T1 cells irradiated with different laser intensities (0, 0.5, 1, 1.25, and 1.5 W/cm^2^) (*n* = 5, mean ± SD). **(C)** Cellular uptake of ASNPs. 4T1 cells coincubated with FITC-labeled ASNPs for 4 or 6 h. Scale bar = 20 μm. **(D)** Confocal images of 4T1 cells stained with DCFH-DA in each group. Scale bar = 20 μm.

First, the biocompatibility of ASNPs was evaluated. From Figure 5A, ASNPs showed good biocompatibility in the absence of NIR irradiation, and the viability of 4T1 cells remained above 95% when the concentration of ASNPs reached 100 ppm. Under NIR irradiation, ASNPs exhibited a good cell-killing effect with IC_50_ of approximately 50 ppm. Next, the cytotoxicity of NIR was evaluated, and almost no cytotoxicity was observed with NIR irradiation alone (Figure 5B). Figure 5C shows the endocytosis ability of 4T1 cells of FITC-labeled ASNPs. ASNPs were endocytosed into cells after cocultured for 4 h, and a high concentration level was still maintained in cells at 6 h. DCFH-DA was used as the ROS fluorescent probe to detect the PEDT process at the cell level. Compared with the other three groups, the intensity of ROS signaled by green fluorescence (DCFH-DA) was significantly increased in 4T1 cells treated with ASNPs + NIR (Figure 5D). The quantitative changes of ROS intensity were further statistically analyzed (Supplementary Figure S5). It can be seen that the fluorescence intensity of the ASNPs + NIR group was 3.84, 5.65, and 4.41 times stronger than that of the control, NIR, and ASNPs groups, respectively, showing the same trend as CLSM images of ROS.

### Therapeutic Efficacy of ASNPs *In Vivo*

The biosafety of ASNPs was evaluated before its *in vivo* treatment applications. There were no abnormalities in body weight, organ sections, and blood indexes of mice in each group, indicating the good biosafety of ASNPs (Supplementary Figures S6–S8).

**FIGURE 6:**
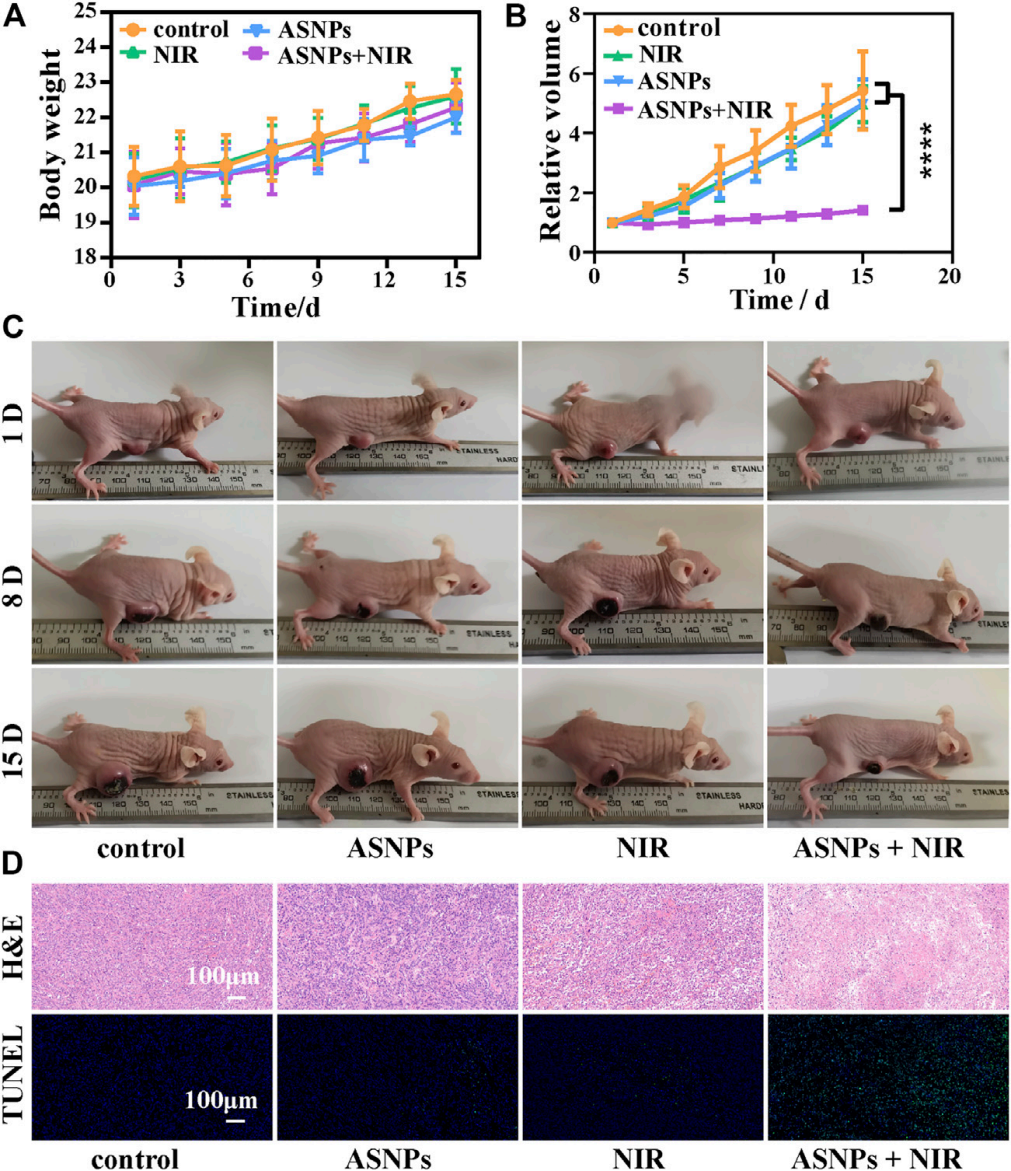
*In vivo* PTT and PEDT performance of ASNPs. **(A)** Body weights, **(B)** relative tumor volumes, and **(C)** photos of mouse with different treatments (15 mg/kg for *i. v.* injection, 808-nm NIR laser: 1 W/cm^2^, 5 min) in the therapeutic process. **(D)** H&E and TUNEL-stained tumor tissues in different treatment groups. (*n* = 5, mean ± SD; ****p < 0.0001).

During treatment, no obvious body weight changes were observed among the control, ASNPs (*i.v.*, 15 mg/kg), NIR (808-nm laser, 1 W/cm^2^, 5 min), and NIR + ASNPs (*i.v.*, 15 mg/kg; 808-nm laser, 1 W/cm^2^, 5 min) groups (Figure 6A). From the statistical results of relative tumor volume changes in each group and images of each group of mice 15 days after treatment (Figures 6B,C), the ASNPs + NIR group exhibited the best tumor growth inhibition performance, whereas the other three groups showed almost no tumor inhibition effect and with little differences. H&E and TUNEL-stained tumor tissue (Figure 6D) showed that cells in the control, ASNPs, and NIR groups hardly suffered any damage, whereas cells in the ASNPs + NIR group showed obvious typical apoptosis, indicating the tremendous tumor cell-killing effect of PEDT–PTT synergetic therapy.

## Conclusion

In summary, ASNPs with great photothermal conversion ability, thermoelectric effect, and carriers’ separation ability were constructed. On the one hand, the ASNPs could convert 808-nm laser into heat for tumor PTT. On the other hand, the ASNPs could produce free e^–^ and h^+^ by responding thermoelectric effect generated by the temperature difference in the photothermal process for direct PEDT to kill tumor cells. Moreover, modified Au-NPs could attract free e^–^ to accumulate, thereby promoting the separation of e^–^ and h^+^ during the PEDT process, significantly increasing ∙OH productivity through e^–^-rich Au-NPs-induced H_2_O_2_ homolysis and h^+^–H_2_O half-reaction. In addition, we speculate that Au-NPs may enhance the affinity between ASNPs and proteins through Au–S interaction, causing an immediate reaction of ∙OH on the HSPs to inhibit the heat resistance of the HSPs. In general, the constructed ASNPs realized the synergistic application of PTT and PEDT in tumor therapy, and the heterostructure material design strategy provided a new method for improving PEDT efficiency by promoting carriers’ separation. This work not only solved the bottleneck problem of PEDT by compositing electrophilic Au-NPs onto SnSe to separate e^–^ and h^+^ but also inspired the researchers to treat temperature difference as a new external energy to trigger specific intracellular reactions. We believe that this work is of great significance for tumor hyperthermia, and will extend its in-depth application in RFA, HIFU, and other biomedical applications.

## Data Availability

The original contributions presented in the study are included in the article/Supplementary Material, further inquiries can be directed to the corresponding author.
