# Supplementary material for: Electron Transfer Strategies to Regulate Carriers’ Separation for Intensive Pyroelectric Dynamic Therapy With Simultaneous Photothermal Therapy
Source: Front Chem. 2022 Apr 12;10:874641. doi: 10.3389/fchem.2022.874641 (PMC9039012; doi:10.3389/fchem.2022.874641)
Supplement: Supplementary file 1 [file DataSheet1.docx]

Supplementary Material


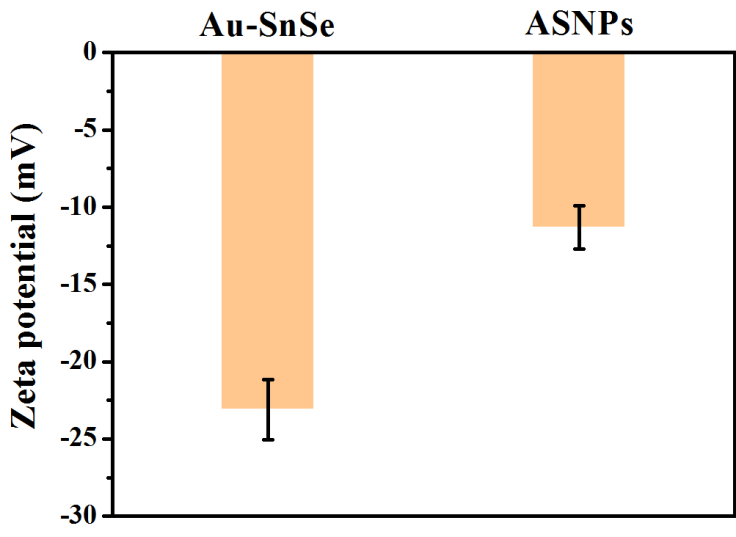


**Supplementary Figure 1.** Zeta-potential of Au-SnSe and ASNPs in DI water.


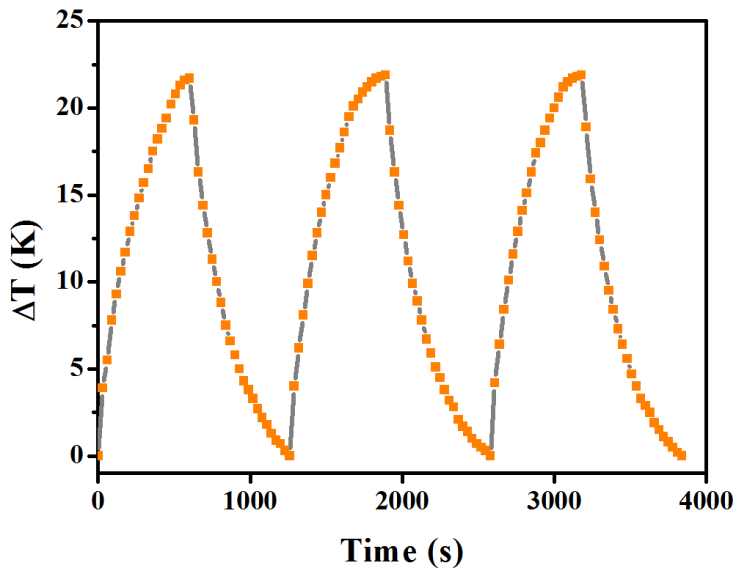


**Supplementary Figure 2.** Heating and cooling cycle curve of ASNPs (50 µg/mL) under irradiation of 808-nm laser (1 W/cm^2^).


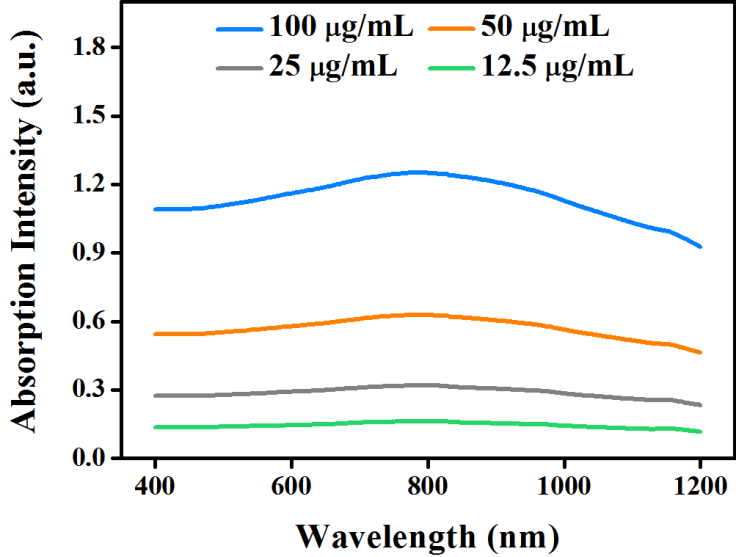


**Supplementary Figure 3.** UV-Vis-NIR absorbance of ASNPs at various concentrations.


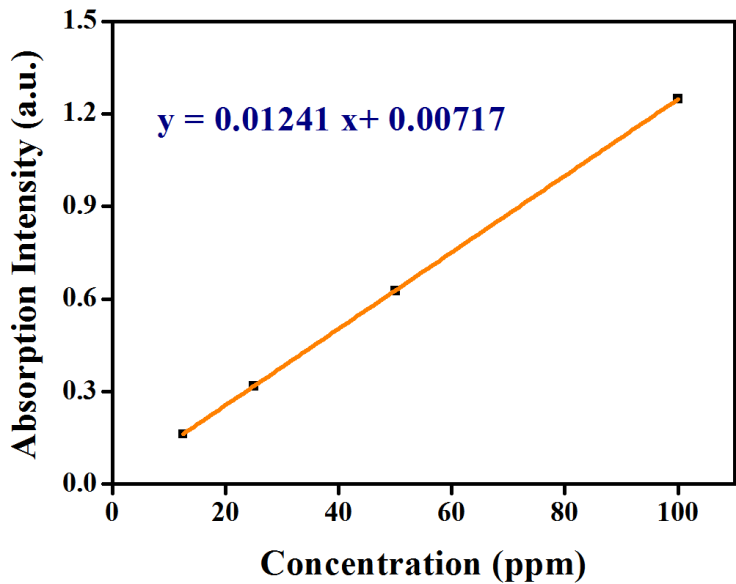


**Supplementary Figure 4.** Scatter plot of absorbance as a function of the concentration of ASNPs.


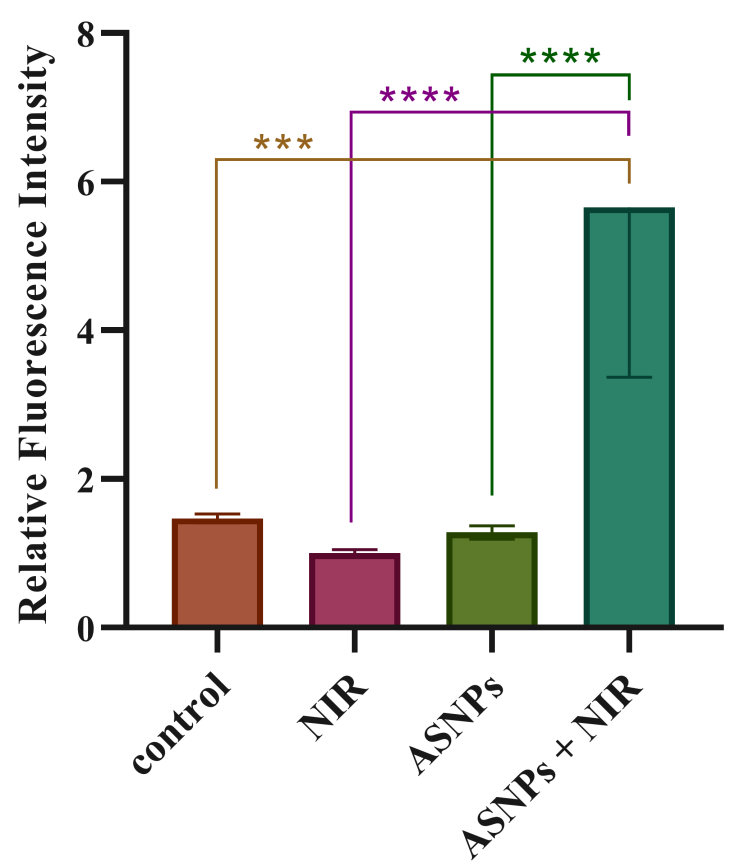


**Supplementary Figure 5.** ROS relative fluorescence intensity of 4T1 cells stained with DCFH-DA in each group. n = 5, mean ± SD, ***p = 0.0001,****p<0.0001.


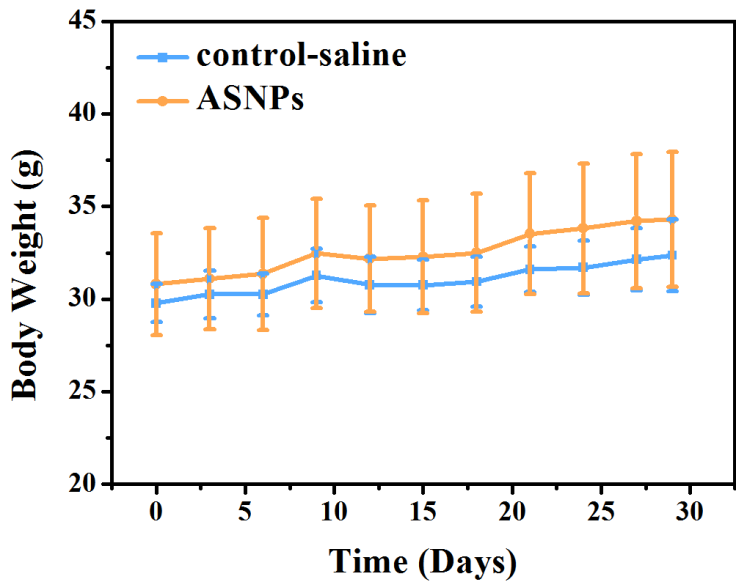


**Supplementary Figure 6.** Time-dependent bodyweight of ICR mice treated with *i.v.* injection of saline solution (100 μL) and ASNPs (30 mg/kg, 100 μL). n = 5, mean ± SD.


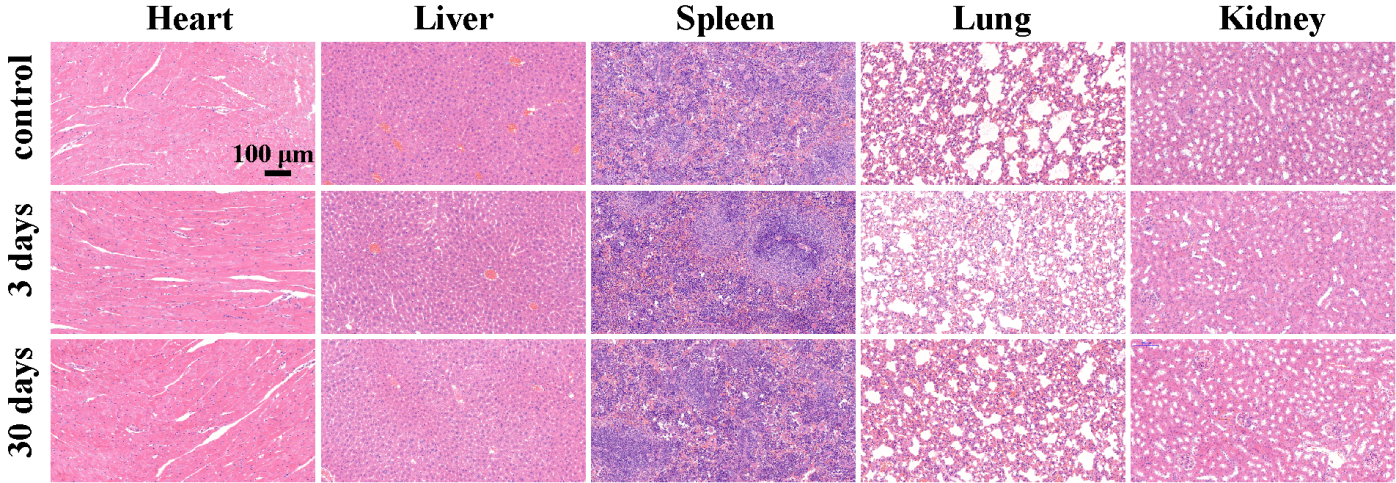


**Supplementary Figure 7.** H&E-stained tissue sections of major organs (heart, liver, spleen, lung, kidney) of ICR mice on the 3rd and 30th day after *i.v.* injection of ASNPs (30 mg/kg, 100 μL), with saline solution *i.v.* injection (100 μL) as control. Scale bar = 100 μm.


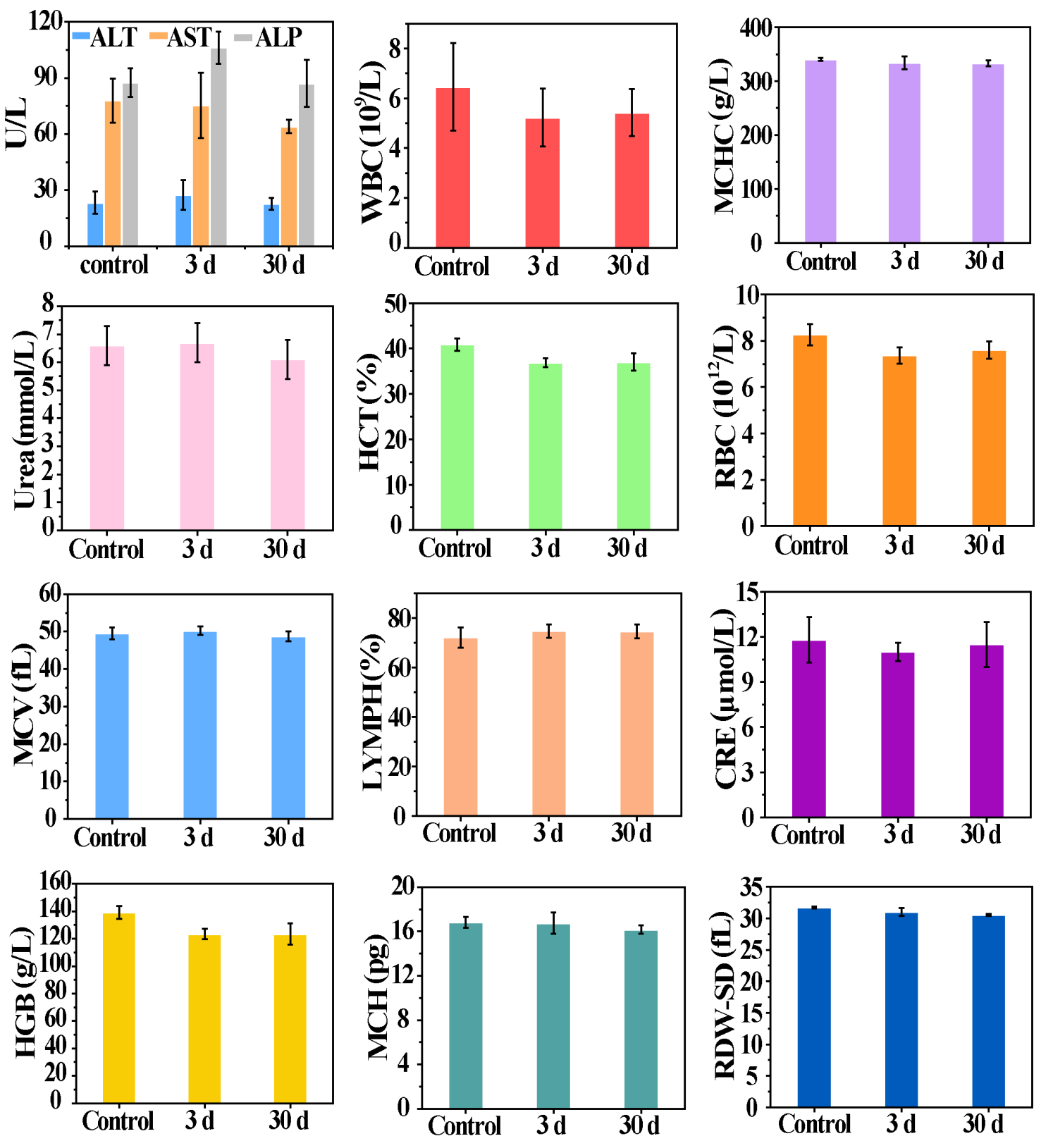


**Supplementary Figure 8.** Blood biochemical indexes and hematology parameters of ICR mice on the 3rd and 30th day after *i.v.* injection of ASNPs (30 mg/kg, 100 μL), with saline solution *i.v.* injection (100 μL) as control. n = 4, mean ± SD.
